# Supplementary material for: Intermittent exposure to high ambient heat during the second half of gestation in mice causes mild alterations of reproductive endpoints in male embryos
Source: bioRxiv. 2026 May 26:2026.05.22.727256. Preprint. [Version 1] doi: 10.64898/2026.05.22.727256 (PMC13232274; doi:10.64898/2026.05.22.727256)

bioRxiv preprint doi: https://doi.org/10.64898/2026.05.22.727256; this version posted May 26, 2026. The copyright holder for this preprint (which was not certified by peer review) is the author/funder. This article is a US Government work. It is not subject to copyright under 17 USC 105 and is also made available for use under a CC0 license.

Supplemental Figure 1: Intermittent gestational heat exposure to 40°C during the second half of pregnancy led to high maternal mortality. (A) Percentage of dams that died during the exposure period.

Supplemental Figure 2: Heat exposure did not alter maternal hormone levels. A-F. Serum levels of corticosterone (A) aldosterone (B) testosterone (C) dihydrotestosterone (D) estradiol (E) or progesterone (F) in dams at E18.5. Each dot represents a single dam. Bar height represents the mean and error bars represent the SEM. Statistical significance was measured using an unpaired t-test. ns, not significant.

bioRxiv preprint doi: https://doi.org/10.64898/2026.05.22.727256; this version posted May 26, 2026. The copyright holder for this preprint (which was not certified by peer review) is the author/funder. This article is a US Government work. It is not subject to copyright under 17 USC 105 and is also made available for use under a CC0 license.

Supplemental Figure 1: Intermittent gestational heat exposure to 40°C during the second half of pregnancy led to high maternal mortality.

A.

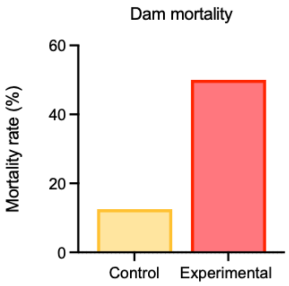

bioRxiv preprint doi: https://doi.org/10.64898/2026.05.22.727256; this version posted May 26, 2026. The copyright holder for this preprint (which was not certified by peer review) is the author/funder. This article is a US Government work. It is not subject to copyright under 17 USC 105 and is also made available for use under a CC0 license.

Supplemental Figure 2: Heat exposure did not alter maternal hormone levels.

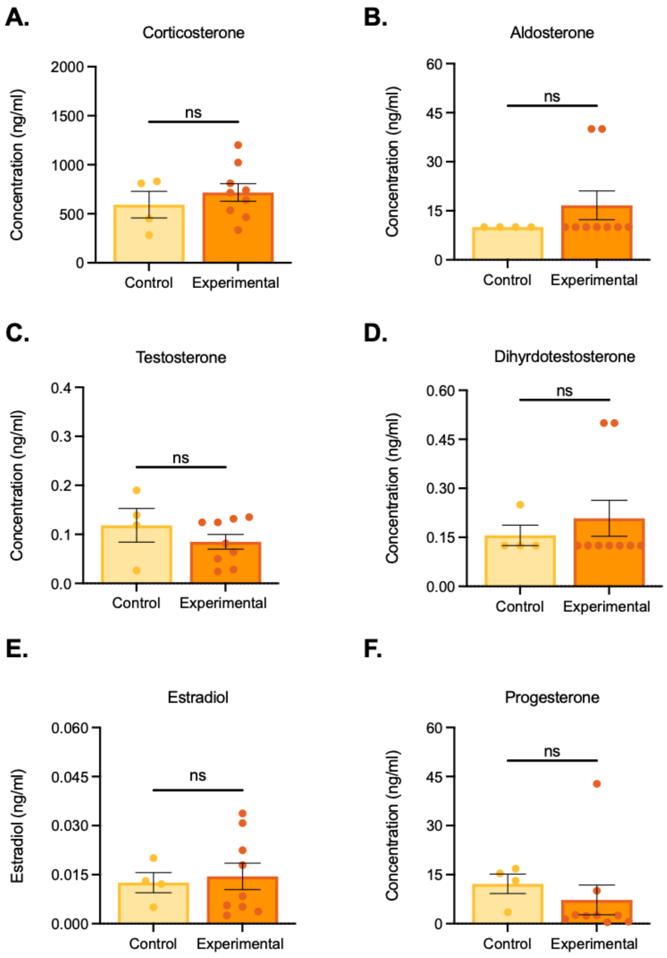

Supplement: Supplement 1 [file NIHPP2026.05.22.727256v1-supplement-1.pdf]
